# Clinical and resource burden of acute kidney injury among adults hospitalized with sepsis: a retrospective cross-sectional study

**DOI:** 10.1186/s12882-026-04915-z

**Published:** 2026-03-24

**Authors:** Brent Tai, Chijioke Okonkwo

**Affiliations:** Baycare Health System, 3231 McMullen Booth Rd, Safety Harbor, Fl 34695 USA

**Keywords:** Sepsis, Acute kidney injury, Discharge disposition, Hospital charges, Length of stay, ICD-10-CM codes, In-hospital mortality, Mechanical ventilation, Central venous catheter placement

## Abstract

**Background:**

Acute kidney injury (AKI) is a frequent complication of sepsis and is associated with worse clinical outcomes. However, contemporary national estimates of its broader clinical and health-system burden, including discharge disposition, remain limited. We used a large nationally representative database to characterize the epidemiology and associated outcomes of AKI among adults hospitalized with sepsis in the United States.

**Methods:**

We conducted a cross-sectional study of adult sepsis hospitalizations in the 2022 National Inpatient Sample (NIS). AKI was identified using ICD-10-CM codes (N17.x) in any diagnosis position. Clinical outcomes included in-hospital mortality, mechanical ventilation, central venous catheter placement (marker of intensive care–level management), and in-hospital dialysis. Resource outcomes included length of stay, total hospital charges, and discharge disposition. Survey-weighted logistic and gamma regression models were used to estimate adjusted odds ratios (aORs) and adjusted ratios of geometric means (aROMs), adjusting for demographic, socioeconomic, and Elixhauser comorbidity variables. Sensitivity analyses evaluated dialysis associations after excluding patients with pre-existing end-stage renal disease (ESRD) and assessed robustness of AKI definition using primary-diagnosis restriction.

**Results:**

Among an estimated 2.96 million adult sepsis hospitalizations in 2022, 44.5% involved AKI. Compared with sepsis hospitalizations without AKI, those with AKI demonstrated higher unadjusted mortality (19.8% vs. 7.5%), greater use of mechanical ventilation (22.3% vs. 9.3%), and increased central venous catheter placement (10.1% vs. 5.8%). After adjustment, AKI remained strongly associated with in-hospital mortality (aOR 2.44; 95% CI 2.38–2.50), mechanical ventilation (aOR 2.66; 95% CI 2.60–2.72), central venous catheter placement (aOR 1.70; 95% CI 1.65–1.74), longer length of stay (aROM 1.33; 95% CI 1.32–1.34), and higher total hospital charges (aROM 1.50; 95% CI 1.48–1.52). AKI was also associated with lower likelihood of home discharge (26.0% vs. 43.8%) and greater transfer to post-acute care facilities (40.4% vs. 30.8%). In fully adjusted models, AKI was associated with lower odds of dialysis (aOR 0.73; 95% CI 0.70–0.76); however, exclusion of patients with pre-existing ESRD reversed this association (aOR 19.0; 95% CI 14.1–25.7), suggesting influence of administrative coding structure. Given potential misclassification of chronic dialysis status in administrative data, dialysis findings should be interpreted as exploratory.

**Conclusions:**

In a contemporary nationally representative cohort, AKI was common among adults hospitalized with sepsis and was independently associated with higher mortality, greater concurrent organ-support involvement, prolonged hospitalization, increased financial burden, and worse discharge disposition. Sensitivity analyses highlight the influence of chronic dialysis coding on dialysis outcomes and underscore the importance of cautious interpretation of administrative data. These findings support recognition of AKI as a high-risk prognostic phenotype within sepsis hospitalizations and emphasize its substantial recovery and health-system burden.

**Supplementary Information:**

The online version contains supplementary material available at 10.1186/s12882-026-04915-z.

## Background

Sepsis remains one of the leading causes of hospitalization, critical illness, and mortality worldwide, contributing to substantial health-care utilization and long-term morbidity [13, 16]. Acute kidney injury (AKI) is among the most frequent and severe organ dysfunctions occurring during sepsis, reported in up to 40–50% of hospitalized patients in prior cohorts and significantly worsening prognosis [9]. The development of AKI in sepsis has been consistently associated with higher mortality, greater need for organ support, prolonged hospitalization, and increased risk of long-term kidney dysfunction [1, 3, 11]. Despite advances in sepsis recognition and management, the burden of sepsis-associated AKI continues to rise and remains a major clinical challenge.

Most prior epidemiologic studies evaluating sepsis-associated AKI have focused on single-center cohorts, ICU-based populations, or older administrative datasets predating current ICD-10 coding practices. These limitations restrict contemporary generalizability and may underestimate the evolving severity and complexity of sepsis-associated AKI. Although the relationship between AKI and mortality or organ-support needs is well established, relatively few studies have evaluated downstream functional outcomes such as discharge disposition—an increasingly important marker of survivorship and recovery. Many landmark analyses, including large multicenter critical care cohorts, reported mortality and organ failure trajectories but did not characterize post-acute disposition patterns [5, 7]. Understanding these broader outcomes is essential to fully quantify the impact of AKI across the entire trajectory of sepsis care.

Using the most recent nationally representative data from the Healthcare Cost and Utilization Project National Inpatient Sample (HCUP NIS), this study aimed to provide an updated and comprehensive assessment of the burden of AKI among adults hospitalized with sepsis in the United States. Specifically, we aimed to (1) describe the national epidemiology of sepsis-associated AKI, (2) quantify clinical and resource outcomes among sepsis hospitalizations with and without AKI, and (3) evaluate whether AKI remains independently associated with adverse outcomes after adjustment for demographic, socioeconomic, and clinical factors.

## Methods

### Study design and data source

We conducted a retrospective cross-sectional study using the 2022 Healthcare Cost and Utilization Project National Inpatient Sample (HCUP NIS), a nationally representative, all-payer inpatient database [2]. The NIS is discharge-based; therefore, each record reflects a single hospitalization. Adult hospitalizations (≥ 18 years) with complete age and survey design information were included. Inter-hospital transfers were retained per HCUP methodology. No human subjects were involved in this study. IRB requirement was waived by Baycare Institutional Review Board.

### Identification of sepsis and acute kidney injury

Sepsis hospitalizations were identified using ICD-10-CM codes A40.x–A41.x (sepsis), R65.20 (severe sepsis), and R65.21 (septic shock). Acute kidney injury (AKI) was defined using ICD-10-CM codes N17.x in any diagnosis position. In sensitivity analyses, AKI was additionally restricted to the primary diagnosis position to assess robustness of exposure classification. Because the NIS lacks present-on-admission indicators, sepsis and AKI may represent either pre-existing conditions or complications arising during hospitalization. Pre-existing end-stage renal disease (ESRD) or chronic dialysis dependence was identified using ICD-10-CM codes N18.6 and Z99.2 in any diagnosis position and was used in sensitivity analyses evaluating dialysis outcomes.

### Baseline characteristics

Baseline demographic, socioeconomic, and comorbidity variables were extracted to compare sepsis hospitalizations with and without AKI. Covariates included age, sex, race/ethnicity, primary payer, ZIP-code income quartile, and Elixhauser comorbidities. Elixhauser comorbidities were derived from ICD-10-CM diagnosis codes using the validated Quan adaptation of the Elixhauser Comorbidity Index. Age was summarized as mean (SD), and categorical variables as survey-weighted percentages. Standardized mean differences (SMDs) were used to quantify between-group differences (SMD ≥ 0.10 indicating meaningful imbalance). These variables correspond to the cohort characteristics presented in Table 1.

### Unadjusted clinical and resource outcomes

Unadjusted outcomes included in-hospital mortality, mechanical ventilation, central venous catheter placement, in-hospital dialysis, length of stay, total hospital charges, and discharge disposition. Mechanical ventilation and dialysis were identified using ICD-10-PCS procedure codes. Central venous catheter placement was identified using ICD-10-PCS procedure codes and analyzed as a marker of intensive care–level management rather than a direct measure of vasopressor administration.

**Fig. 1:**
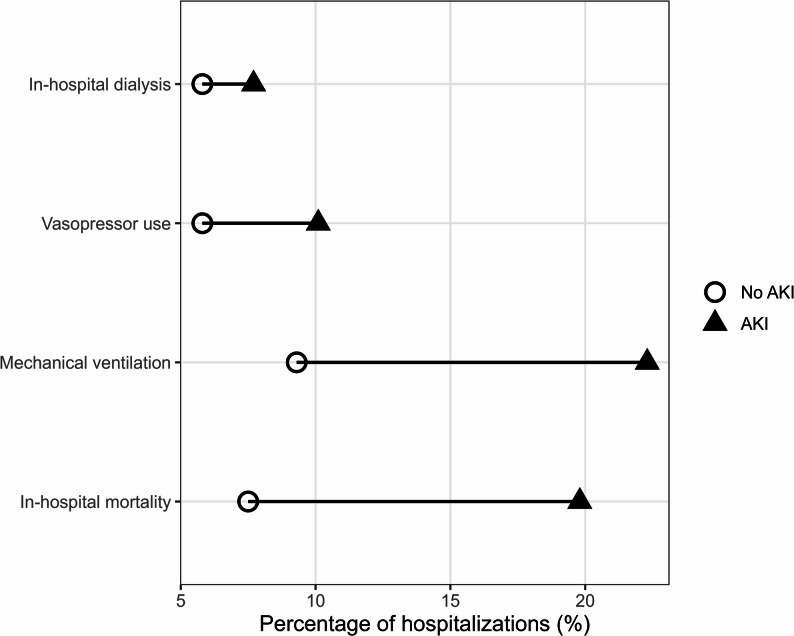
Clinical outcomes among sepsis hospitalizations with and without acute kidney injury

**Fig. 2:**
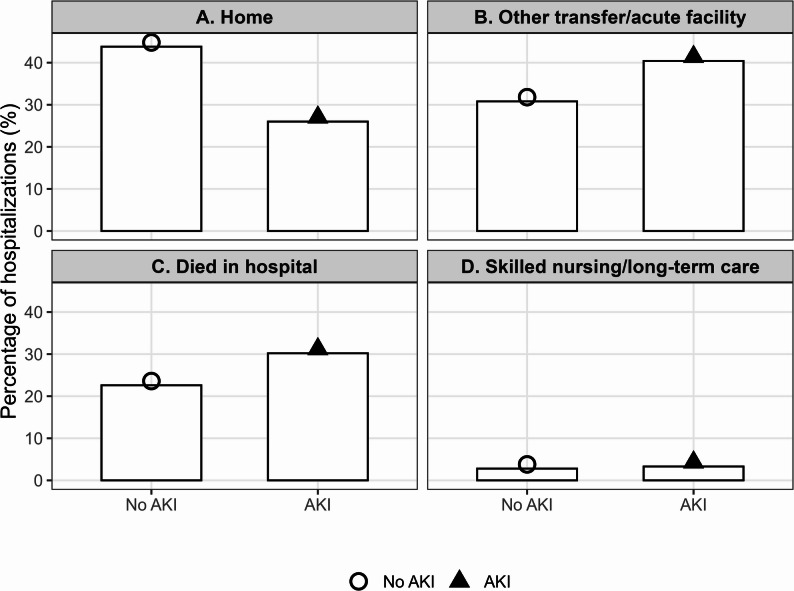
Distribution of discharge disposition by acute kidney injury status among adults hospitalized with sepsis

Discharge disposition was categorized as home, transfer to another acute-care facility, skilled nursing/long-term care, or in-hospital death. Because mortality (DIED) and discharge disposition derive from different NIS fields, the proportion “died in hospital” differs from the in-hospital mortality variable. Unadjusted clinical outcomes presented in Table 2. They are visually summarized in Fig. 1, and discharge disposition patterns are displayed in Fig. 2.

### Adjusted analyses

**Fig. 3:**
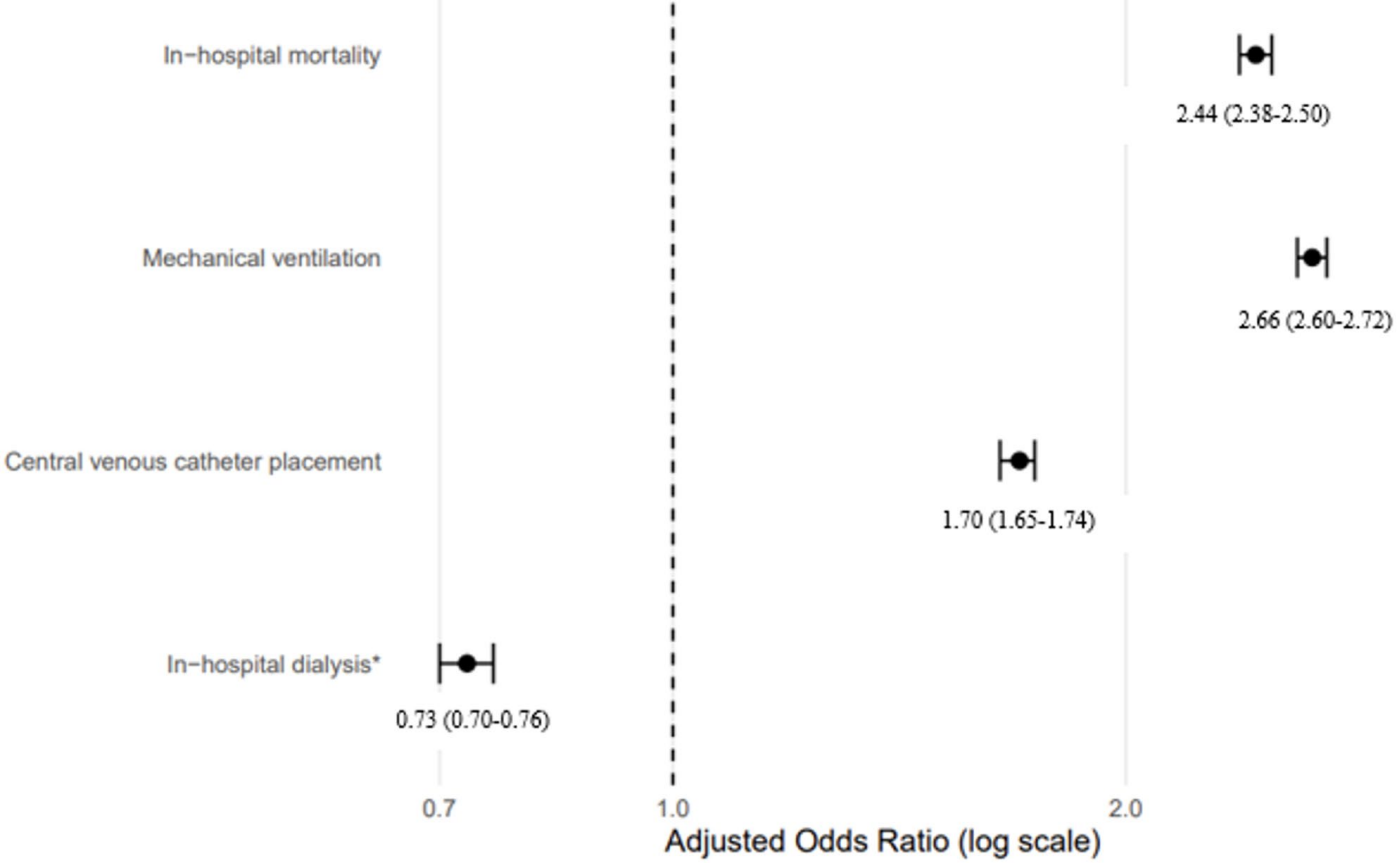
Adjusted associations between acute kidney injury and clinical outcomes among adults hospitalized with sepsis

The independent association between AKI and each outcome was evaluated using multivariable survey-weighted regression. Binary outcomes—including mortality, mechanical ventilation, central venous catheter placement, and dialysis—were analyzed using logistic regression. Given potential misclassification of dialysis procedures in patients with pre-existing ESRD, dialysis findings were interpreted cautiously and evaluated in sensitivity analyses. Length of stay and total hospital charges, which were right-skewed, were analyzed using gamma regression with a log link and reported as adjusted ratios of geometric means (aROMs). All models adjusted for demographic, socioeconomic, and comorbidity variables listed in Table 1. The adjusted associations are presented in Table 3 and visually depicted in Fig. 3.

### Sensitivity analyses

Several prespecified sensitivity analyses were conducted. First, to evaluate the robustness of the dialysis findings, models were re-estimated excluding patients with pre-existing ESRD or dialysis dependence. Second, to assess the influence of chronic renal failure adjustment, models were re-estimated excluding the Elixhauser chronic renal failure (rf) variable. Third, AKI was restricted to the primary diagnosis position to assess exposure specificity in relation to mortality outcomes. All sensitivity analyses used the same survey-weighted modeling approach as the primary analyses.

### Statistical analysis

All analyses applied NIS sampling weights, strata, and clusters to produce nationally representative estimates. Continuous variables were summarized using weighted means or medians as appropriate; categorical variables using weighted percentages. Analyses were performed using R version 4.3 or later (survey, srvyr, tidyverse, broom, ggplot2). Single–primary sampling unit (PSU) strata were handled using the “adjust” option within the survey package in accordance with HCUP analytic recommendations.

## Results

Among an estimated 2.96 million adult sepsis hospitalizations in 2022, 1.32 million (44.5%) were complicated by acute kidney injury (AKI). Patients with AKI were older (mean age 67.9 vs. 62.6 years) and had substantially higher prevalence of chronic kidney disease, heart failure, diabetes with complications, and coagulopathy (all SMD ≥ 0.20). They were also more frequently insured through Medicare (64.4% vs. 55.5%). Differences in race distribution were modest (SMD = 0.10), and ZIP-code income quartiles were similar between groups. Baseline characteristics are shown in Table 1.

**Table 1:** Baseline Characteristics of Adult Sepsis Hospitalizations

Characteristic	Sepsis without AKI	Sepsis + AKI	SMD
Unweighted count	327,836	264,940	
Weighted count	1,639,180	1,321,700	
**Demographics**			
Age, years	62.6 (17.9)	67.9 (15.3)	0.32
Female, %	50.1	44.9	0.11
**Comorbidities**			
Chronic kidney disease / renal failure, %	17.6	38.0	0.47
Chronic pulmonary disease, %	27.6	25.5	0.047
Coagulopathy, %	11.6	21.0	0.26
Diabetes with chronic complications, %	24.3	33.8	0.21
Diabetes without chronic complications, %	10.6	8.6	0.066
Heart failure, %	22.9	34.8	0.27
Hypertension, complicated, %	27.2	46.6	0.41
Hypertension, uncomplicated, %	34.4	25.0	0.21
Liver disease, %	9.3	16.0	0.20
Solid tumor (nometastasis), %	4.7	4.9	0.008
Metastatic cancer, %	5.5	5.7	0.012
Obesity, %	19.8	20.9	0.027
Peripheral vascular disease, %	7.7	9.5	0.067
**Payer type**			0.19
Medicaid	17.5	13.4	
Medicare	55.5	64.4	
Missing	0.1	0.1	
No charge	0.4	0.2	
Other	2.9	2.9	
Private insurance	19.5	16.1	
Self-pay	4.1	2.9	
**Race**			0.10
Asian	3.2	3.0	
Black	13.2	15.1	
Hispanic	13.4	10.6	
Missing	2.1	2.3	
Native American	0.9	0.8	
Other	2.8	2.8	
White	64.4	65.4	
**ZIP income**,** percentile**			0.021
0–25th	30.0	30.7	
26–50th	25.7	25.5	
51–75th	23.4	23.3	
76–100th	19.3	19.0	
Missing	1.7	1.5	

Table 1 summarizes the baseline demographic, socioeconomic, and comorbidity characteristics of adults hospitalized with sepsis, comparing those with and without acute kidney injury. Age is expressed as mean (standard deviation). All other variables are presented as weighted percentages. Unweighted counts represent the raw NIS sample size, whereas weighted counts represent national estimates. Standardized mean differences (SMD) quantify the magnitude of differences between groups, with values ≥ 0.10 generally indicating meaningful imbalance.

Patients with AKI experienced substantially worse clinical outcomes than those without AKI. Compared with non-AKI hospitalizations, those with AKI demonstrated higher in-hospital mortality (19.8% vs. 7.5%), mechanical ventilation use (22.3% vs. 9.3%), central venous catheter placement (10.1% vs. 5.8%), and dialysis use (7.7% vs. 5.8%). Unadjusted outcomes are presented in Table 2. These differences are visually depicted in Fig. 1, which highlights the substantially greater organ-support needs associated with AKI.

**Table 2:** Clinical outcomes of adult sepsis hospitalizations

Outcome	Sepsis without AKI	Sepsis + AKI	SMD
**Clinical Outcomes**			
In-hospital mortality, %	7.5	19.8	0.36
Mechanical ventilation, %	9.3	22.3	0.35
Central venous catheter placement, %	5.8	10.1	0.16
In-hospital dialysis, %	5.8	7.7	0.076
**Resource Utilization**			
Length of stay, median (IQR), days	5.0 (3.0–9.0)	7.0 (4.0–13.0)	0.25
Total hospital charges, median (IQR), $	$55,757 ($30,756–$108,313)	$84,698 ($43,633–$178,705)	
**Discharge Disposition**,** %**			0.38
Died in hospital	22.6	30.2	
Home	43.8	26.0	
Other transfer/acute facility	30.8	40.4	
Skilled nursing/long-term care	2.8	3.3	

Table 2 summarizes unadjusted clinical outcomes, resource utilization, and discharge disposition among adults hospitalized with sepsis, stratified by AKI status. Continuous measures reported as median (IQR); categorical variables as weighted percentages. SMD ≥ 0.10 indicates meaningful imbalance.

Figure 1 displays the unadjusted clinical outcomes of sepsis hospitalizations stratified by AKI status, illustrating higher mortality and greater organ-support needs among patients with AKI. Shapes represent AKI status.

AKI hospitalizations had significantly greater resource use: length of stay (7.0 vs. 5.0 days) and hospital charges ($84,698 vs. $55,757). Discharge outcomes also differed markedly. Home discharge was less common (26.0% vs. 43.8%), whereas transfer to post-acute care was more frequent (40.4% vs. 30.8%). The proportion categorized as “died in hospital” differs from in-hospital mortality due to separate NIS coding fields. Accordingly, percentages reported under discharge disposition are not directly comparable to the binary in-hospital mortality outcome. Figure 2 illustrates these disposition patterns, demonstrating a clear shift toward higher post-acute care needs among patients with AKI.

Figure 2 illustrates the distribution of discharge dispositions among sepsis hospitalizations, showing that patients with AKI were less likely to be discharged home and more likely to transfer to acute or post-acute facilities. Discharge disposition categories include home (A), other acute care transfer (B), died in hospital (C), and skilled nursing/long-term care (D). Bars represent percentages; shapes denote AKI status.

After adjustment for demographic, socioeconomic, and comorbidity factors, AKI remained strongly associated with in-hospital mortality (aOR 2.44; 95% CI 2.38–2.50), mechanical ventilation (aOR 2.66; 95% CI 2.60–2.72), central venous catheter placement (aOR 1.70; 95% CI 1.65–1.74), and in-hospital dialysis (aOR 0.73; 95% CI 0.70–0.76) (Table 3). These adjusted associations are visually summarized in Fig. 3. AKI was also associated with greater resource utilization, including longer length of stay and higher total hospital charges.

**Table 3:** Adjusted Association of Acute Kidney Injury with Clinical and Resource Outcomes Among Adults Hospitalized with Sepsis

Outcome	aOR / aROM (95% CI)	*p*-value
**Clinical Outcomes**		
In-hospital mortality	2.44 (2.38–2.50)	< 0.001
Mechanical ventilation	2.66 (2.60–2.72)	< 0.001
Central venous catheter placement	1.70 (1.65–1.74)	< 0.001
In-hospital dialysis	0.73 (0.70–0.76)	< 0.001
**Resource Utilization**		
Length of stay	1.33 (1.32–1.34)	< 0.001
Total hospital charges	1.50 (1.48–1.52)	< 0.001

Table 3 presents the adjusted associations between AKI and key clinical and resource outcomes among adults hospitalized with sepsis. Binary outcomes were reported as adjusted odds ratios (aORs). Length of stay and total hospital charges were reported as adjusted ratios of geometric means (aROM). All models adjust for demographics, payer and income factors, and comorbidities.

Figure 3 provides a visual summary of the adjusted associations between acute kidney injury and key in-hospital clinical outcomes, illustrating the magnitude and precision of effect estimates relative to sepsis hospitalizations without AKI. Points represent adjusted odds ratios (aORs) estimated from survey-weighted logistic regression models, and horizontal bars indicate 95% confidence intervals. The reference group is sepsis hospitalizations without acute kidney injury 1.

Because dialysis findings differed from other outcomes, additional sensitivity analyses were performed (Supplementary Table 1). In minimally adjusted models including age and sex only, AKI was associated with higher odds of dialysis (OR 1.40; 95% CI 1.36–1.45). In the fully adjusted model excluding the Elixhauser chronic renal failure (rf) variable, the association attenuated toward the null (aOR 0.87; 95% CI 0.84–0.91).

Importantly, after excluding patients with pre-existing end-stage renal disease (ESRD) or dialysis dependence (ICD-10-CM codes N18.6 or Z99.2), the association between AKI and dialysis reversed direction and became strongly positive (aOR 19.04; 95% CI 14.12–25.68). These findings suggest that the inverse association observed in the primary fully adjusted model was driven by inclusion of chronic dialysis patients and reflects administrative coding structure rather than a biologically protective relationship.

Additional sensitivity analysis restricting AKI to the primary diagnosis position yielded similar associations with in-hospital mortality (aOR 2.14; 95% CI 1.92–2.38) compared with the primary definition using any diagnosis position (aOR 2.44; 95% CI 2.38–2.50) (Supplementary Table 2), supporting robustness of the exposure definition.

## Discussion

While prior studies have documented increased mortality and organ support among patients with sepsis-associated AKI, fewer analyses have examined discharge disposition and post-acute care transitions at a national level. By incorporating discharge trajectories and resource utilization in a contemporary national cohort, this study expands understanding of the broader recovery and health-system burden associated with AKI in sepsis.

In this nationally representative analysis of nearly three million adult sepsis hospitalizations in the United States, acute kidney injury (AKI) emerged as a common and clinically consequential complication. Almost half of all sepsis admissions experienced AKI, and these hospitalizations demonstrated substantially worse outcomes across multiple domains. Patients with sepsis-associated AKI had markedly higher in-hospital mortality, greater involvement of mechanical ventilation and central venous catheter placement, and increased use of in-hospital dialysis compared with those without AKI. These adverse clinical differences were accompanied by significantly longer hospital stays, higher total charges, and a greater likelihood of discharge to post-acute or long-term care facilities. Together, these findings highlight the substantial clinical and recovery burden associated with AKI in sepsis.

Our findings align closely with prior observational studies demonstrating that AKI substantially worsens outcomes in sepsis. Prior analyses from large administrative datasets similarly report two- to threefold higher mortality and greater organ support needs among patients with sepsis-associated AKI [1, 4, 17]. The adjusted associations illustrated in Fig. 3 demonstrate that AKI remains strongly associated with mortality and concurrent organ-support involvement even after extensive adjustment for demographic and comorbidity factors.

Prospective cohorts have shown that the development of AKI in sepsis reflects underlying illness severity and contributes to progression of multiorgan failure [6, 11]. Studies using earlier versions of the HCUP NIS have documented increased hospitalization costs and prolonged length of stay in sepsis hospitalizations complicated by AKI [14]. However, few prior investigations have examined post-acute discharge disposition in detail. By incorporating these trajectories, our study broadens the understanding of the recovery burden associated with AKI, demonstrating that its impact extends well beyond the index hospitalization. Collectively, our findings reinforce AKI as a strong prognostic marker of illness severity within sepsis hospitalizations. While temporality cannot be established in administrative data, the consistent associations observed across multiple outcomes underscore the importance of recognizing AKI as a high-risk clinical phenotype.

Several mechanisms likely underlie the strong association between AKI and adverse outcomes in sepsis observed in this study. Sepsis triggers a cascade of systemic inflammation, endothelial injury, microvascular dysfunction, and hemodynamic instability, all of which predispose the kidney to ischemic and toxic insults [10]. Conversely, AKI may amplify systemic inflammation, impair metabolic and acid–base homeostasis, and contribute to fluid overload, thereby worsening pulmonary and cardiovascular function [3].

AKI hospitalizations were more likely to involve mechanical ventilation and central venous catheter placement, suggesting greater overall illness severity and multiorgan dysfunction. Because temporal sequencing cannot be determined in the National Inpatient Sample, these interventions should be interpreted as concurrent markers of critical illness rather than downstream consequences of AKI. The increased reliance on post-acute or long-term care services among survivors with AKI likely reflects the broader impact of prolonged critical illness, impaired functional recovery, and persistent organ dysfunction. Together, these mechanisms underscore how AKI serves as a marker of severity within the broader syndrome of multiorgan dysfunction in sepsis.

The inverse association between AKI and dialysis observed in the fully adjusted model warrants careful interpretation. In minimally adjusted analyses, AKI was associated with higher odds of dialysis, consistent with biological plausibility. However, in fully adjusted models that included chronic renal failure and other comorbidities, the association attenuated and reversed direction. Sensitivity analyses demonstrated that exclusion of patients with pre-existing end-stage renal disease (ESRD) or dialysis dependence reversed the association and yielded markedly higher odds of dialysis among AKI hospitalizations. These findings suggest that the inverse association is largely influenced by inclusion of chronic dialysis patients and administrative coding structure rather than a biologically protective effect of AKI. Accordingly, dialysis findings should be interpreted as exploratory and subject to coding limitations inherent to administrative data.

The clinical and health-system implications of these findings are substantial. Because AKI in sepsis is both common and strongly linked to a broad spectrum of adverse outcomes, early identification and mitigation of kidney injury should be a central priority in sepsis management. Evidence-based strategies—including careful fluid stewardship, avoidance of nephrotoxins, and hemodynamic optimization—have been proposed to mitigate AKI risk in critically ill populations [8]. The greater lengths of stay, hospital charges, and post-acute care needs associated with AKI are clearly reflected in Fig. 2, highlighting the system-level burden imposed by this complication. Interventions aimed at mitigating AKI may therefore yield meaningful clinical, functional, and economic benefit across the spectrum of sepsis care.

This study has several notable strengths. It uses the most recent iteration of the HCUP National Inpatient Sample, providing a large, nationally representative cohort of adults hospitalized with sepsis and enabling robust estimation of both clinical and resource outcomes. The use of survey-weighted methods ensures proper accounting for the complex sampling design, enhancing generalizability to U.S. inpatient care. We applied standardized approaches to identifying sepsis, AKI, comorbidities, and organ-support interventions, and adjusted for a comprehensive set of demographics, socioeconomic, and clinical covariates. Importantly, this analysis extends prior work by incorporating discharge disposition as an outcome, offering additional insights into the functional and recovery burden associated with sepsis-associated AKI.

Several limitations should be considered when interpreting these findings. First, this study relied on administrative claims data, and identification of sepsis, AKI, mechanical ventilation, central venous catheter placement, and dialysis was based on ICD-10-CM/PCS coding rather than clinical measurements. Although these codes have been used extensively in prior epidemiologic research [12], they may be subject to misclassification and cannot distinguish between conditions present on admission and those acquired in the hospital. The absence of laboratory data precluded staging of AKI using serum creatinine or urine output criteria, limiting granular assessment of kidney injury severity [15].

Second, as with all observational studies, residual confounding is possible despite adjustment for key demographic, socioeconomic, and comorbidity variables. The NIS lacks detailed information on illness severity, medication exposures, and clinical management decisions, all of which may influence the development of AKI and downstream outcomes. Furthermore, discharge disposition does not capture post-discharge functional recovery or long-term survival. Finally, because the NIS is discharge-based rather than patient-based, repeated admissions cannot be linked, and results reflect hospitalizations rather than individuals. These limitations notwithstanding, the consistency and magnitude of the observed associations support the robustness of our conclusions.

Because identification of AKI relied on ICD-10-CM codes, we conducted additional sensitivity analyses restricting AKI to the primary diagnosis position. The association between AKI and in-hospital mortality remained strong and statistically significant under this restrictive definition, supporting robustness of the exposure classification. Although administrative coding may underestimate milder stages of AKI, the consistency of mortality associations across definitions suggests that misclassification is unlikely to account for the primary findings.

## Conclusion

In summary, acute kidney injury remains a common and clinically consequential complication among adults hospitalized with sepsis. AKI hospitalizations are characterized by substantially higher mortality, greater concurrent organ-support involvement, prolonged hospitalization, increased financial burden, and worse discharge disposition. These findings highlight AKI as a high-risk prognostic phenotype within sepsis hospitalizations and underscore the need for improved risk stratification and recovery planning in this population.

## Supplementary Information

Below is the link to the electronic supplementary material.

Supplementary Material 1

Supplementary Material 2

## Data Availability

The data that support the findings of this study are available from the Agency for Healthcare Research and Quality, Department of Health and Human Services of the United States. However, restrictions apply to the availability of these data, which were used under license for the current study, and so are not publicly available. Data are however available from the author upon reasonable request and with permission of the Agency for Healthcare Research and Quality.

## Footnotes

1 * In-hospital dialysis findings are interpreted as exploratory due to potential misclassification of chronic dialysis status; see Supplementary Table 1 for sensitivity analyses.

## Declarations

### AI assistance

During the preparation of this work, Large Language Models (LLMs) were not used. The authors reviewed and revised the material and took full responsibility for the content.
