# Supplementary material for: Clinical and resource burden of acute kidney injury among adults hospitalized with sepsis: a retrospective cross-sectional study
Source: BMC Nephrol. 2026 Mar 24;27:277. doi: 10.1186/s12882-026-04915-z (PMC13137707; doi:10.1186/s12882-026-04915-z)
Supplement: Supplementary file 1 — Supplementary Material 1 [file 12882_2026_4915_MOESM1_ESM.docx]

Supplementary Table 1. Sensitivity analyses of the association between acute kidney injury and in-hospital dialysis among adults hospitalized with sepsis

| **Model Specification** | **AKI aOR** | **95% CI** | **p-value** |
| --- | --- | --- | --- |
| Model 1: Age + sex adjusted | 1.40 | 1.36–1.45 | <0.001 |
| Model 2: Fully adjusted (primary model) | 0.73 | 0.70–0.76 | <0.001 |
| Model 3: Fully adjusted, excluding chronic renal failure (rf) | 0.87 | 0.84–0.91 | <0.001 |
| Model 4: Fully adjusted, excluding ESRD/dialysis-dependent patients | 19.04 | 14.12–25.68 | <0.001 |

Supplementary Table 1 presents sensitivity analyses evaluating the association between acute kidney injury and in-hospital dialysis under alternative model specifications, including exclusion of patients with pre-existing end-stage renal disease and removal of the chronic renal failure covariate. Fully adjusted models include age, sex, race, ZIP income quartile, primary payer, and Elixhauser comorbidities (congestive heart failure, chronic pulmonary disease, diabetics with/without chronic complication, renal failure, lung disease, obesity, coagulopathy, solid tumors, metastatic cancer, peripheral vascular disease, hypertension with/without complications). ESRD was defined by ICD-10-CM codes N18.6 or Z99.2. Survey-weighted logistic regression with HCUP-recommended handling of single-PSU strata was used.
