# Supplementary material for: Clinical and resource burden of acute kidney injury among adults hospitalized with sepsis: a retrospective cross-sectional study
Source: BMC Nephrol. 2026 Mar 24;27:277. doi: 10.1186/s12882-026-04915-z (PMC13137707; doi:10.1186/s12882-026-04915-z)
Supplement: Supplementary file 2 — Supplementary Material 2 [file 12882_2026_4915_MOESM2_ESM.docx]

Supplementary Table 2. Sensitivity analysis restricting acute kidney injury to primary diagnosis position

| **Model** | **AKI Definition** | **aOR** | **95% CI** | **p-value** |
| --- | --- | --- | --- | --- |
| Fully adjusted mortality model | AKI in any diagnosis field | 2.44 | 2.38–2.50 | <0.001 |
| Fully adjusted mortality model | AKI in primary diagnosis only | 2.14 | 1.92–2.38 | <0.001 |

Supplementary Table 2 evaluates the robustness of the primary findings by restricting acute kidney injury to the primary diagnosis position, thereby assessing whether the observed association with in-hospital mortality persists under a more specific exposure definition. AKI was defined using ICD-10-CM N17.x codes in the primary diagnosis position only. Adjusted odds ratios (aORs) were estimated from survey-weighted logistic regression models adjusting for demographic, socioeconomic, and Elixhauser comorbidity variables.
